# Supplementary figures and images for: African Gene Flow Reduces Beta-Ionone Anosmia/Hyposmia Prevalence in Admixed Malagasy Populations
Source: Brain Sci. 2021 Oct 25;11(11):1405. doi: 10.3390/brainsci11111405 (PMC8615941; doi:10.3390/brainsci11111405)

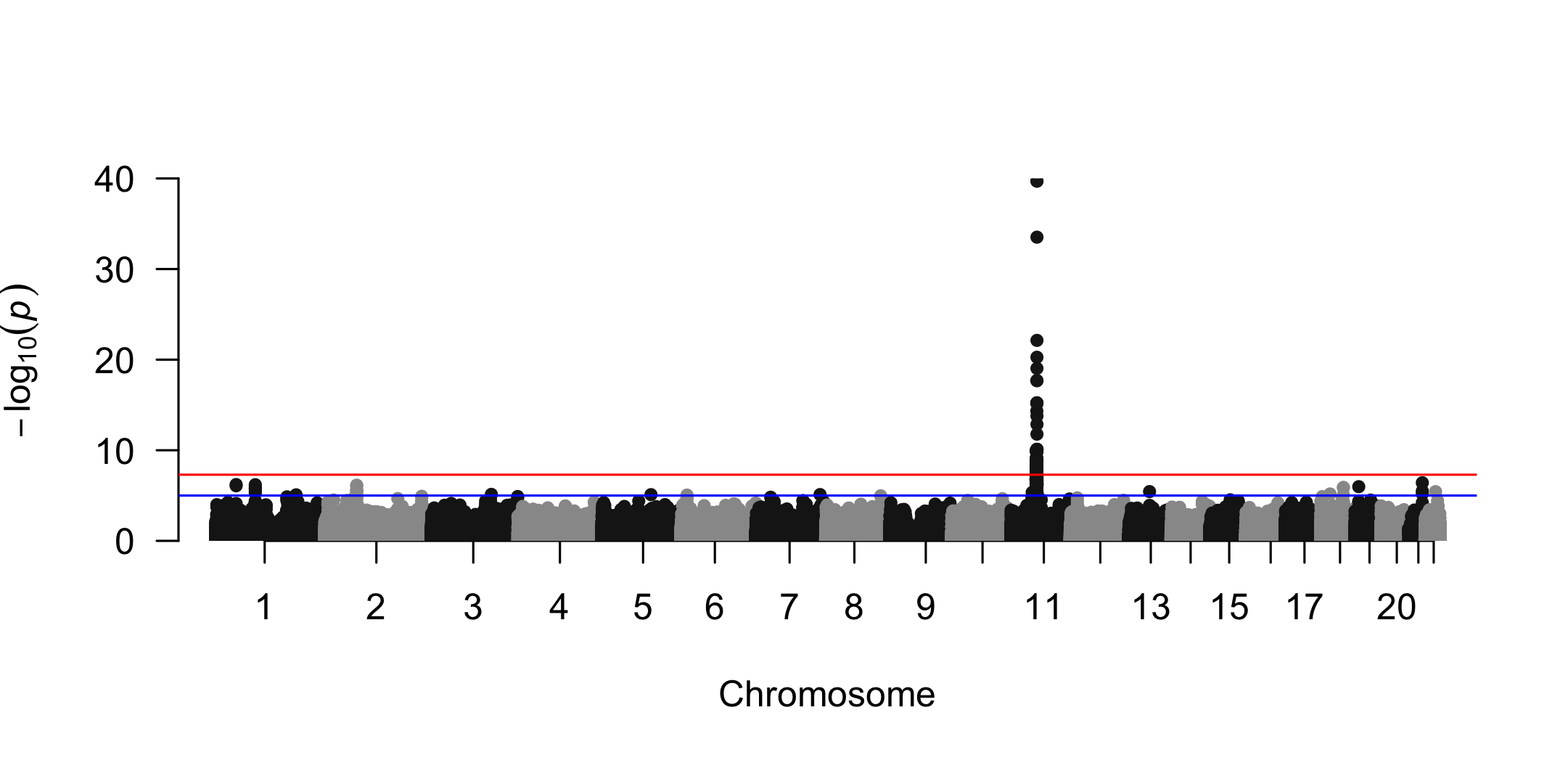

Supplement: Supplementary file 1 [file brainsci-11-01405-s001.zip › Figure S1.png]
